# ALK5 i II Accelerates Induction of Adipose-Derived Stem Cells toward Schwann Cells through a Non-Smad Signaling Pathway

**DOI:** 10.1155/2021/8307797

**Published:** 2021-10-15

**Authors:** Seiji Sawai, Tsunao Kishida, Shin-ichiro Kotani, Shinji Tsuchida, Ryo Oda, Hiroyoshi Fujiwara, Kenji Takahashi, Osam Mazda, Yoshihiro Sowa

**Affiliations:** ^1^Department of Orthopedics, Kyoto Prefectural University of Medicine, Kamigyo, Kyoto 602-8566, Japan; ^2^Department of Immunology, Kyoto Prefectural University of Medicine, Kamigyo, Kyoto 602-8566, Japan; ^3^Department of Orthopedics, Japanese Red Cross Society Kyoto Daini Hospital, Kyoto 602-8026, Japan; ^4^Department of Plastic and Reconstructive Surgery, Kyoto Prefectural University of Medicine, Kamigyo, Kyoto 602-8566, Japan

## Abstract

Schwann cells (SCs) are likely to be a vital component of cell-based therapies for nerve regeneration. There are various methods for inducing SC-like cells (SCLCs) from adipose-derived stem cells (ADSCs), but their phenotypic and functional characteristics remain unsatisfactory. Here, we report a novel efficient procedure to induce SCLCs by culturing ADSCs with ALK5 inhibitor (ALK5 i) II, a specific inhibitor of activin-like kinase 5 (ALK5) (transforming growth factor-*β* receptor 1 (TGF*β*R1)) that is also known as Repsox. The resultant cells that we named “modified SCLCs (mSCLCs)” expressed SC-specific genes more strongly than conventional SCLCs (cSCLCs) and displayed a neurosupportive capacity in vitro, similarly to genuine SCs. Regarding the mechanism of the mSCLC induction by ALK5 i II, knockdown of Smad2 and Smad3, key proteins in the TGF*β*/Smad signaling pathway, did not induce SC markers. Meanwhile, expression of multipotent stem cell markers such as Sex-determining region Y- (SRY-) box 2 (Sox2) was upregulated during induction. These findings imply that ALK5 i II exerts its effect via the non-Smad pathway and following upregulation of undifferentiated cell-related genes such as Sox2. The procedure described here results in highly efficient induction of ADSCs into transgene-free and highly functional SCLCs. This approach might be applicable to regeneration therapy for peripheral nerve injury.

## 1. Introduction

Treatment of peripheral nerve injuries focuses on application of Schwann cells (SCs), which are promising candidates for cell-based therapies, including transplantation. However, this procedure has significant drawbacks in clinical application, including the main disadvantage of sacrifice of a functional nerve to produce autologous SCs. Therefore, methods to generate sufficient SCs using alternative advanced stem cell technologies are being examined. These include lineage-specific differentiation and reprogramming to generate SCs from various cell types [1–3]. Among the cell sources, adipose-derived stem cells (ADSCs) are promising due to their ease of collection in sufficient amounts through a minimally invasive procedure. Rat ADSCs can be induced into SC-like cells (SCLCs) [4–6], and this method can be applied to human ADSCs. However, SCLCs derived from human ADSCs have low expression of SC markers such as S100*β* [7]. S100*β* is expressed in SCs and acts as a stimulator of cell proliferation and migration. S100*β* also functions an inhibitor of apoptosis and differentiation [8–10].

Somatic cell reprogramming is basically triggered by transduction of the genes that encode important transcription factors, while an addition of some small molecular compounds may modify the reprogramming processes. Applications of TGF*β* receptor (TGF*β*R) inhibitors [11, 12], a MEK-ERK pathway inhibitor [11–13], and a GSK3 inhibitor [13] have been shown to improve efficacy of generation of induced pluripotent stem (iPS) cells. Moreover, one or more of the Yamanaka factors were successfully replaced by TGF*β*R inhibitors [14] and a GSK3 inhibitor [15, 16]. Direct conversion of fibroblasts into transgene-free osteoblasts or adipocytes can also be achieved with small molecules [17, 18].

Here, we found that a TGF*β*R inhibitor induced ADSCs to exhibit SC-like phenotypes, such as expression of S100*β*, GAP43, and EGR2. The mSCLCs promoted neurite outgrowth more strongly than conventional SCLCs (cSCLCs) and to a similar extent to genuine SCs (gSCs). We also investigated the induction mechanism by means of knockdown of specific signaling molecules.

## 2. Materials and Methods

### 2.1. Reagents and Medium

The small molecules used in this study are listed in Supplementary Tables S1 and S2. The complete medium consisted of Dulbecco's modified Eagle's medium (DMEM) supplemented with 100 mM nonessential amino acids, 100 U/ml penicillin, 100 *μ*g/ml streptomycin, and 10% fetal bovine serum (FBS). This medium was supplemented with 5 ng/ml platelet-derived growth factor (PDGF; Wako, Osaka, Japan), 10 ng/ml basic fibroblast growth factor (b-FGF; Wako), 5.7 *μ*g/ml forskolin (TCI, Tokyo, Japan), and 200 ng/ml recombinant human heregulin-*β*1 (HRG-*β*1; R&D Systems, Minneapolis, MN, USA) and used as a SC medium.

### 2.2. Antibodies

The following primary antibodies were used for immunocytostaining: rabbit anti-S100*β* (dilution = 1 : 200) (Abcam, Cambridge, UK), rabbit anti-GAP43 (1 : 200) (Cell Signaling Technology, Danvers, MA, USA), rabbit anti-EGR2 (1 : 200) (Abcam), rabbit anti-NCAM (1 : 200) (Sino Biological, Beijing, China), and mouse anti-MAP-2 (1 : 200) (Abcam) antibodies. Alexa Fluor 488-conjugated anti-rabbit (1 : 500) (Life Technologies, Carlsbad, CA, USA) and Alexa Fluor 594-conjugated anti-mouse (1 : 500) (Life Technologies) antibodies were used as secondary antibodies. For western blotting, rabbit anti-Smad2/3 (dilution = 1 : 1000) (Cell Signaling Technology), rabbit anti-phospho Smad2/3 (1 : 5000) (Cell Signaling Technology), and rabbit anti-*β*-actin (1 : 5000) (Cell Signaling Technology) antibodies were used. For flow cytometric analysis, phycoerythrin- (PE-) conjugated mouse anti-human CD29 (dilution = 1 : 25) (BioLegend, San Diego, CA, USA), Alexa Fluor 488-conjugated rat anti-mouse/human CD44 (1 : 25) (BioLegend), PE-conjugated mouse anti-human CD90 (1 : 5) (BD Biosciences, San Jose, CA, USA), and fluorescein isothiocyanate- (FITC-) conjugated mouse anti-human CD105 (1 : 25) (BioLegend) antibodies were used.

### 2.3. Isolation and Culture of Human ADSCs

Adipose tissue was harvested from female surgical patients at the University Hospital, Kyoto Prefectural University of Medicine, Japan. Patients gave informed consent, and procedures were approved by the Institutional Ethics Committee for Clinical Research at Kyoto Prefectural University of Medicine (ERB-C-487-1). A previously described protocol was used for isolation and culture of ADSCs, with a slight modification [4]. Briefly, subcutaneous human fat was enzymatically dissociated at 37°C for 60 min using 0.15% (*w*/*v*) collagenase type I (Sigma-Aldrich, St. Louis, MO, USA). The solution was passed through a 70 *μ*m filter (Corning, Corning, NY, USA) to remove undissociated tissue and neutralized by the addition of a complete medium. The stromal cell pellet was obtained by centrifugation for 5 min and resuspended in the complete medium. After 24 h, the nonadherent cells were eliminated by changing the medium. Cultures were maintained at subconfluent levels in a 37°C incubator with 5% CO_2_ and passaged 2-4 times with trypsin/EDTA (Nacalai Tesque, Kyoto, Japan) before use.

### 2.4. Differentiation of ADSCs into cSCLCs

ADSCs were differentiated into cSCLCs as previously described [4] using HRG-*β*1 instead of glial growth factor 2. Briefly, subconfluent ADSCs at passages 2-4 were cultured in a complete medium containing 1 mM 2-mercaptoethanol (BME, Sigma-Aldrich) for 24 h. The cells were then washed and placed in a fresh medium supplemented with 35 ng/ml all-*trans*-retinoic acid (ATRA, TCI). After 72 h, the cells were washed and the medium was replaced by a SC medium with or without small molecules added at a concentration of 4 *μ*M, unless otherwise stated. The cells were incubated for 14 days to achieve full differentiation, with the medium changed every 2 to 3 days (Figure S1).

### 2.5. Isolation and Culture of gSCs

gSCs were obtained as previously described [7]. Peripheral nerve specimens were taken from patients during reconstructive surgery with informed consent. After removing the epineurium, the nerves were cut to 1 mm segments. The segments were cultured in a 60 mm dish with a SC medium at 37°C with 5% CO_2_. Two weeks later, the medium was aspirated and 0.0625% collagenase type IV and 0.585 U dispase were added to the dish. After a 24 h incubation, the cell suspension was filtered through a 70 *μ*m cell strainer, followed by centrifugation for 5 min. Finally, the cell pellet was resuspended in a SC medium and plated in a 60 mm dish coated with laminin (Matrixome, Osaka, Japan). The cultures were maintained at subconfluent levels in a 37°C incubator with 5% CO_2_.

### 2.6. RNAi

shRNA sequences were found by FASMAC as follows: 5′-CAAGTACTCCTTGCTGGATTG-3′ for Smad2, and 5′-TGAGCAGAACAGGTAGTATTA-3′ for Smad3. Nonsilencing control shRNA sequence (5′-GTTCTCCGAACGTGTCACGT-3′) was confirmed by BLAST analysis (http://blast.ncbi.nlm.nih.gov.ezp.lib.unimelb.edu.au/Blast.cgi) to have no complementarity to any mammalian mRNA sequence. Oligonucleotides were synthesized and inserted into pGP/Puro and pGP/Bsd empty vector plasmids to construct pGP/Puro-Smad2-shRNA (sh-Smad2), pGP/Bsd-Smad3-shRNA (sh-Smad3), and pGP/Puro-negative control-shRNA plasmids (sh-NC). The plasmids were transfected into HEK293 TN cells using the X-tremeGENE 9 DNA Transfection Reagent (Roche Diagnostics GmbH, Mannheim, Germany). Twenty-four hours later, the culture medium was replaced with an antibiotic-free culture medium. After culturing for another 24 hours, the medium was collected and filtrated through a 0.45 mm pore-sized filter. ADSCs were seeded onto culture dishes. After culturing overnight, cells were transduced with lentiviral vectors containing sh-Smad2 and/or lentiviral vectors containing sh-Smad3 in the presence of 4 *μ*g/ml polybrene. Twenty-four hours later, the culture medium was replaced with a complete medium. Stable transfectants were selected by puromycin (for sh-NC and sh-Smad2) and/or blasticidin (for sh-Smad3).

### 2.7. Immunocytochemistry

Cells were fixed in 4% paraformaldehyde (PFA) for 20 min, followed by permeabilization using 1% Triton X-100 at room temperature for 15 min. Background staining was blocked using Blocking One Histo (Nacalai Tesque) for 1 h at room temperature, followed by incubation of the cells with primary antibodies at 4°C overnight. Cells were washed and incubated with secondary antibodies for another 1 h at room temperature. Cells were then washed and counterstained with Hoechst (Wako) for 10 min at room temperature. After washing, cells were mounted and observed under a fluorescence microscope (BZ-X710; Keyence, Osaka, Japan).

### 2.8. Neurite Outgrowth Assay

A neurite outgrowth assay using NG108-15 neuronal cells was performed as previously described, with a slight modification [7]. ADSCs, cSCLCs, mSCLCs, and gSCs were seeded at 6 × 10^3^ cells per slide chamber (Fukae Kasei Co., Ltd., Kobe, Japan) and maintained for 24 h. Then, 6 × 10^2^ NG108-15 neuronal cells were added to the slide chambers, and the cocultures were maintained in DMEM/Ham's F12 plus 2% B27 supplement (Gibco-BRL, Grand Island, NY, USA) for 24 h. The cells were fixed and immunostained with anti-MAP-2 antibody, followed by staining with Alexa Fluor 594-conjugated anti-rabbit antibody, as described above. The chambers were examined under a fluorescence microscope, and neurite analysis was performed using the digital image analysis program ImageJ (public domain). Three parameters were assessed: (1) the percentage of neuron-bearing neurites; (2) the longest neurite length; and (3) the number of neurites per neuron. A total of 80 neuronal cells were examined for each coculture, and the means ± S.D. of three independent experiments for each coculture type was calculated.

### 2.9. Real-Time RT-PCR

Real-time RT-PCR was performed as described elsewhere [19]. Briefly, cells were homogenized in Buffer RLT (Qiagen, Hilden, Germany), and total RNA was harvested using the phenol guanidinium acid-based procedure with QIAcube (Qiagen). After reverse transcription into cDNA using the ReverTra Ace® qPCR RT Master Mix (Toyobo, Osaka, Japan), real-time PCR was performed using the StepOnePlus real-time PCR System (Applied Biosystems, Bedford, MA, USA) with the TaqMan® Fast Advanced Master Mix (Applied Biosystems) and the primers and dye probes shown in Supplementary Table S3. Each 20 *μ*l reaction mixture contained 2 *μ*l of cDNA (100 ng), 1 *μ*l of each primer and probe, and 10 *μ*l of TaqMan® Fast Advanced Master Mix. Samples were incubated at 95°C for 10 min for initial denaturation, followed by 40 cycles of denaturation at 95°C for 15 s, and annealing and extension at 60°C for 1 min. Relative mRNA levels were calculated using the comparative threshold cycle (CT) method and normalized against GAPDH as an internal control.

### 2.10. Western Blotting

Cells were extracted in lysis buffer containing a protease inhibitor cocktail and a phosphatase inhibitor cocktail (Nacalai Tesque) for 1 hour on ice. The lysates were centrifuged at 10,000 rpm for 10 minutes, incubated at 70°C for 10 minutes, and separated by SDS-PAGE (30 *μ*g protein/lane). After transferring to a PVDF membrane using the iBlot 2 system (Life Technology, Carlsbad, CA, USA), the blot was probed with the antibodies described above at 4°C overnight. After 1-hour incubation with an HRP-labeled anti-rabbit immunoglobulin antibody (Cell Signaling Technology) (diluted at 1 : 20,000) at room temperature, signals were visualized using the ECL Select detection reagent (for phosphorylated Smad2/3) (GE Healthcare, Chicago, IL, USA) or ECL Prime detection reagent (for Smad2/3 and *β*-actin) (GE Healthcare) and analyzed by ImageQuant LAS 500 (GE Healthcare).

### 2.11. Flow Cytometric Analysis

Dissociated cells were resuspended in phosphate-buffered saline (PBS) containing 0.5% bovine serum albumin (BSA), 0.01% NaN_3_, and 1 mM EDTA (FACS Buffer) and incubated with antibodies for 20 min on ice. Cells were washed with FACS Buffer, and flow cytometric analysis was performed on FACSCalibur (BD Biosciences) using CellQuest software. The data were analyzed with FlowJo software (Tree Star).

### 2.12. Statistical Analysis

**Figure 1:**
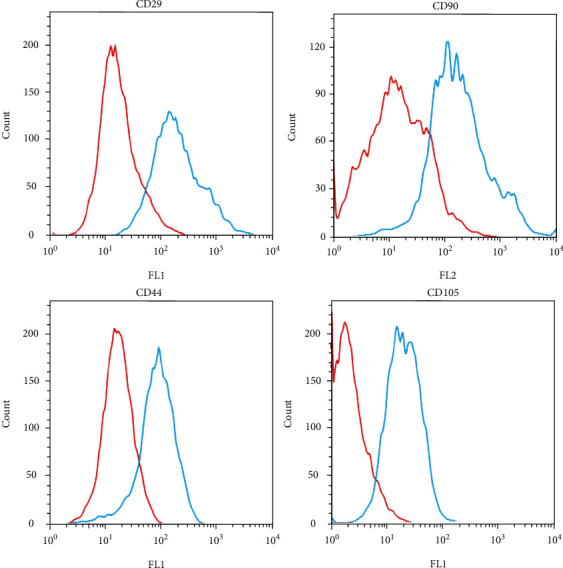
MSC markers decreased upon transition of ADSCs to mSCLCs. Human ADSCs were seeded onto 60 mm dishes and cultured in a complete medium or a SC medium supplemented with ALK5 i II for 14 days. Flow cytometric analysis was performed to examine CD29, CD44, CD90, and CD105 expression on the cell surface. Representative histograms for ADSCs (blue line) and mSCLCs (red line) are shown.

**Figure 2:**
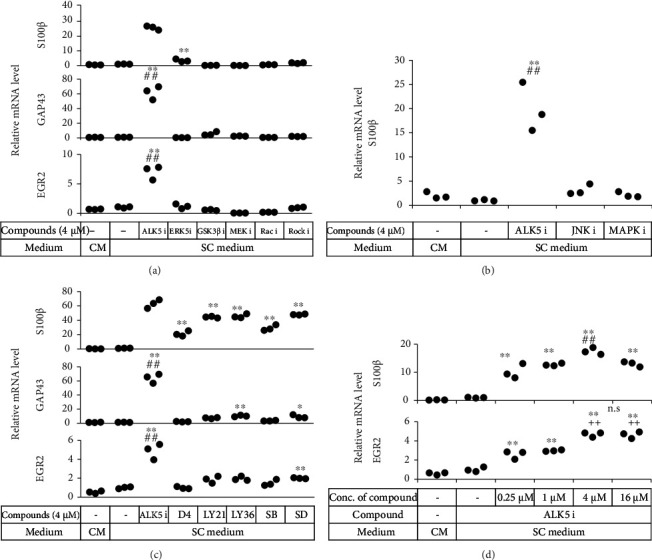
ALK5 i II prompted hADSCs to express SC markers. Human ADSCs were seeded in 24-well plates and cultured in a complete medium or a SC medium supplemented with the compounds indicated. Various signal pathway inhibitors (a, b) and TGF*β*R inhibitors (c) were compared, while various concentrations of ALK5 i II were also tested (d). After 14 days of culture, RNA was extracted from the cells and subjected to real-time RT-PCR analysis. Each dot represents triplicate value of relative mRNA level for the indicated genes. ^∗^*p* < 0.05 and ^∗∗^*p* < 0.01 vs. ADSCs cultured in the SC medium. ^##^*p* < 0.01 vs. all the other groups. ^++^*p* < 0.01 vs. ADSCs cultured in the SC medium supplemented with ALK5 i II at 0, 0.25, or 1 *μ*M. n.s.: no significant difference between the indicated groups. Experiments were repeated three times.

**Figure 3:**
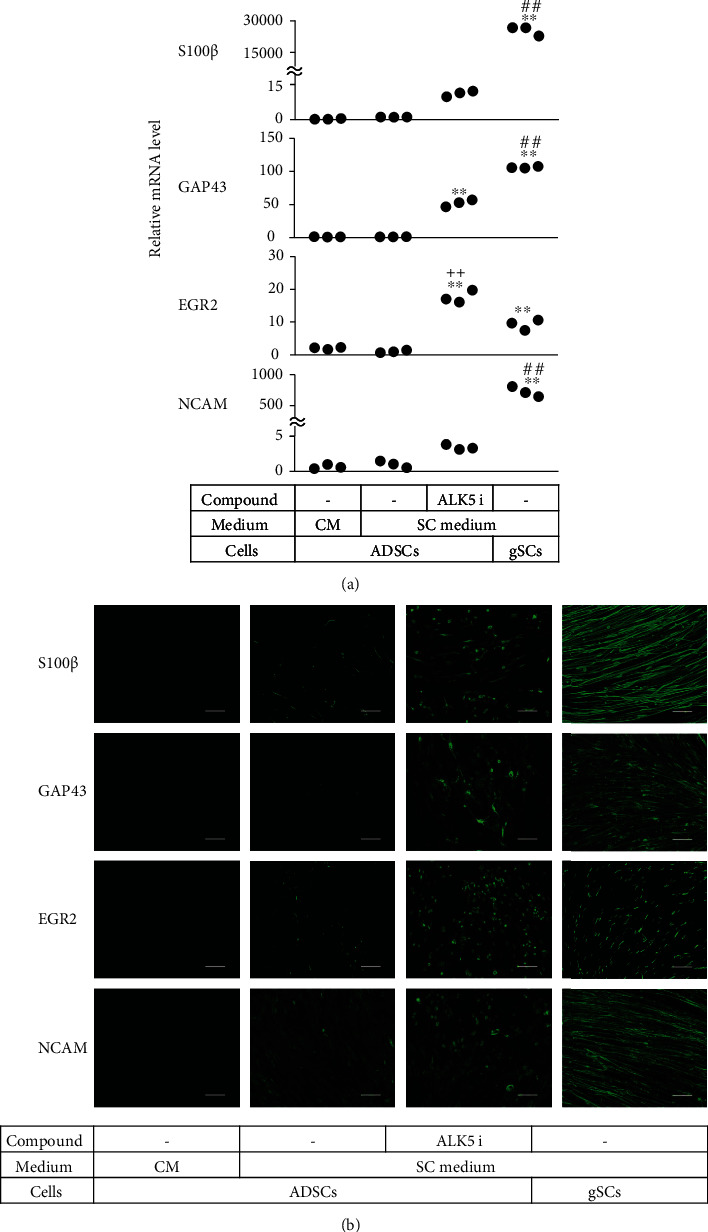
Comparison of relative mRNA levels among ADSCs, cSCLCs, mSCLCs, and gSCs. Cells were seeded into 24-well plates and cultured in the indicated medium for 14 days. (a) RNA was extracted from the cells, and real-time RT-PCR was performed to evaluate mRNAs for the indicated genes. Each dot represents triplicate value. ^∗∗^*p* < 0.01 vs. ADSCs. ^##^*p* < 0.01 vs. mSCLCs. ^++^*p* < 0.01 vs. gSCs. (b) ADSCs as negative control and cSCLCs, mSCLCs, and gSCs as positive control were stained with the indicated antibodies, while cell nuclei were stained with Hoechst. Fluorescence microscopic images (magnification: ×200) are shown. Scale bar = 100 *μ*m. Experiments were repeated three times.

**Figure 4:**
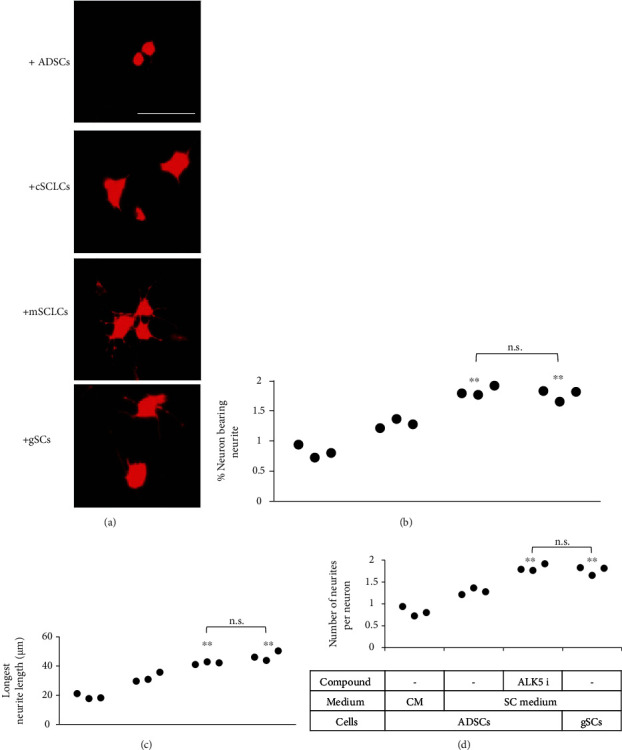
mSCLCs enhanced neurite outgrowth in NG108-15 cells. NG108-15 neuronal cells were cocultured with ADSCs (+ADSCs), cSCLCs (+cSCLCs), mSCLCs (+mSCLCs), or gSCs (+gSCs) for 24 h. NG108-15 cells were immunostained with MAP-2 antibody (red fluorescence) for neurite growth analysis. (a) Representative fluorescence microscopic images are shown. Scale bar = 100 *μ*m. (b–d) Percentages of neurons bearing neurites (b), longest neurite lengths (c), and number of neurites per neuron (d) were calculated. Each dot represents a triplicate value. ^∗∗^*p* < 0.01 vs. ADSCs cultured in a SC medium alone. n.s.: no significant difference between the indicated groups.

**Figure 5:**
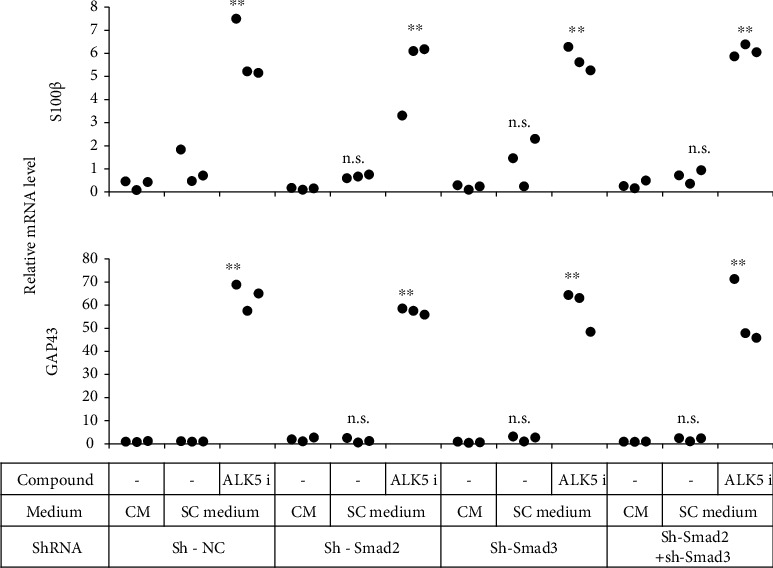
Neither Smad2 nor Smad3 were indispensable for induction of ADSCs into mSCLCs. ADSCs were transduced with lentiviral vectors encoding sh-NC, sh-Smad2, and/or sh-Smad3 as indicated. Forty-eight hours later, selection of transfected cells was started by adding puromycin and/or blasticidin. Stable transfectants were treated with BME and ATRA as indicated and cultured in the indicated culture medium as in Figure S1. After 14 days of culture, RNA was extracted from the cells and subjected to real-time RT-PCR to determine mRNA levels of the indicated genes. Each dot represents a triplicate value. ^∗∗^*p* < 0.01 vs. sh-NC. n.s.: no significant difference between the indicated groups.

**Figure 6:**
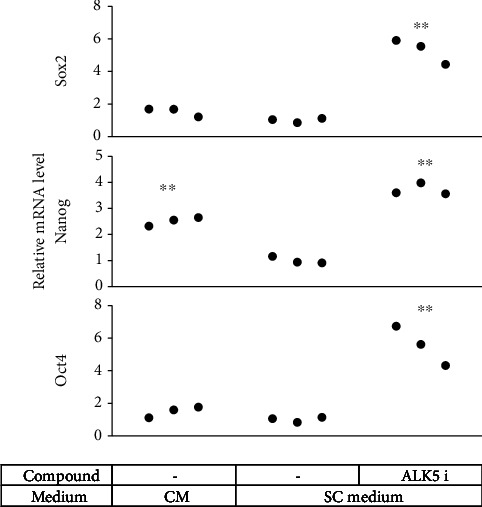
Impact of ALK5 i II on pluripotency-related gene expression in mSCLCs. ADSCs were cultured in a SC medium with ALK5 i II as in Figure 2. RNA was extracted from the cells 3 days after medium replacement (Figure S1) and subjected to real-time RT-PCR to evaluate mRNA levels for the indicated genes. Each dot represents a triplicate value of the relative mRNA level. ^∗∗^*p* < 0.01 vs. ADSCs cultured in the SC medium alone.

Statistical significance was analyzed using Student's *t*-test (Figure 1 and Table S4) and ANOVA with the Tukey-Kramer post hoc test (Figures 2–6, Figure S4). Differences were considered significant at *p* < 0.05.

## 3. Results

### 3.1. Induction of Human ADSCs to a SC-Like Phenotype by Treatment with ALK5 i II

Some compounds enhance reprogramming of somatic cells into iPS cells and contribute to the maintenance of stem cell phenotypes, while others promote differentiation of stem cells into somatic cells [11, 13, 20, 21]. Six compounds (ALK5 i II, ERK5 inhibitor, GSK3B inhibitor, MEK inhibitor, Rac inhibitor, and ROCK inhibitor) were assessed for their ability to induce Schwann-like phenotypes in ADSCs. ADSCs were cultured in a SC medium supplemented with each compound for 14 days, and levels of mRNAs for SC-related genes were examined. The TGF*β*R inhibitor ALK5 i II most significantly elevated expression of S100*β*, GAP43, and EGR2 (*p* < 0.01) (Figure 2(a)). We also tested the JNK inhibitor and the p38 MAP kinase inhibitor, and we found that only ALK5 i II significantly elevated expression of S100*β* (*p* < 0.01) (Figure 2(b)).

Next, we compared the abilities of six TGF*β*R inhibitors (ALK5 i II, D4476, LY2157299, LY364947, SB431542, and SD208) to induce expression of SC-related genes. Again, ALK5 i II most significantly elevated expression of S100*β*, GAP43, and EGR2 (*p* < 0.01) (Figure 2(c)). Thus, ALK5 i II was considered to be the most potent compound for induction of SCLCs from ADSCs. Optimal concentration of ALK5 i II for the SCLC induction was 4 *μ*M (Figure 2(d)). Then, the ADSCs cultured in a SC medium with 4 *μ*M ALK5 i II for 14 days were referred to as mSCLCs and used in subsequent experiments.

### 3.2. mSCLCs Expressed SC Markers at Higher Levels than cSCLCs

We tested mSCLCs for expression of mRNA for SC-related genes. Real-time RT-PCR revealed that mSCLCs expressed mRNA for S100*β*, GAP43, EGR2, and NCAM approximately 11-fold, 18-fold, and 3.4-fold higher than cSCLCs, respectively (Figure 3(a)). Immunofluorescence also showed that a larger proportion of mSCLCs strongly expressed these SC markers (Figure 3(b) and Figures S2A–D) compared with cSCLCs. Some mSCLCs displayed bipolar and tripolar morphologies and strongly expressed S100*β* and GFAP proteins. Uninduced ADSCs did not show any expression of S100*β* and GFAP proteins.

Although undifferentiated ADSCs were highly positive for CD29, CD44, CD90, and CD105, the expression levels of these MSC markers decreased upon induction of the ADSCs into mSCLCs by ALK5 i II (Figure 1 and Table S4).

### 3.3. mSCLCs Promote Neurite Outgrowth at a Comparable Level as gSCs

To compare the functions of mSCLCs and cSCLCs, quantitative analysis of neurite outgrowth induction was performed. ADSCs, cSCLCs, mSCLCs, and gSCs were cocultured with a motor neuron-like cell line, NG108-15 neuronal cells [22]. After 24 h, three parameters, i.e., the percentage of neurons bearing neurites, the longest neurite length, and the number of neurites per neuron, were quantified by immunostaining of the neuronal cells with anti-MAP-2 antibody (Figure 4(a)). All the parameters consistently indicated that mSCLCs prompted NG108-15 cells to extend neurites as strongly as gSCs did, and the ability of mSCLCs to induce neurite extension was more significant than that of cSCLCs (Figures 4(b)–4(d)).

### 3.4. Smad Signaling Pathway May Not Be Essentially Involved in the Induction of mSCLCs by ALK5 i II

The mechanism of induction of ADSCs into Schwann-like cells was investigated using shRNA interference. ALK5 i II is a selective TGF*β*R1 inhibitor that inhibits Smad/non-Smad signaling pathways [14, 23]. In the Smad signaling pathway, Smad2 and Smad3 are key proteins located downstream of TGF*β*R1 [24]. Treatment of ADSCs with ALK5 i II inhibited the phosphorylation of Smad2/3 in our experimental settings (Figure S3). Therefore, shRNA was used to inhibit expression of Smad2 and/or Smad3 to investigate the effect of Smad signaling on induction. ADSCs were established in which Smad2 and/or Smad3 expression was selectively decreased using lentiviral shRNA. Each vector enabled selection for shRNA expression, based on puromycin and/or blasticidin resistance. In the selected cells, the efficiencies and specificities of gene silencing were assessed by real-time RT-PCR at day 7 after lentiviral infection. As shown in Figure S4, psh-Smad2 decreased Smad2 mRNA with 91.2% efficiency and psh-Smad3 decreased Smad3 mRNA with 91.5% efficiency. Psh-Smad2 did not affect Smad3 expression and vice versa. Psh-Smad2/psh-Smad3 decreased Smad2 mRNA and Smad3 mRNA each with 91.2% efficiency (Figure S4).

We next evaluated the effects of decreased Smad2 and/or Smad3 expression at day 7 after lentiviral shRNA vector infection. ADSCs with downregulated Smad2 and/or Smad3 were cultured in a Schwann medium supplemented with or without ALK5 i II for 14 days after BME and ATRA treatment, and expression levels of mRNAs for S100*β* and GAP43 were measured. Regardless of downregulation of Smad2 and/or Smad3, ADSCs cultured in the SC medium supplemented with ALK5 i II had elevated expression of S100*β* and GAP43; thus, downregulation of Smad2 and/or Smad3 had no effects on expression of these markers (Figure 5).

### 3.5. Transcription Factor Expression in Pluripotent Stem Cells during Induction

ALK5 i II can replace exogenous Sex-determining region Y- (SRY-) box 2 (Sox2) in reprogramming fibroblasts into iPS cells [25]. Inhibition of TGF*β* signaling is accompanied by induction of other essential pluripotency-related transcription factors such as Nanog [14, 26]. Sox2 is expressed in undifferentiated SCs and is often used as a SC marker. Therefore, we examined expression of these transcription factor genes in ADSCs shortly after ALK5 i II treatment. Expression of Sox2 increased by approximately 5.3-fold relative to that in the cells cultured in the SC medium without ALK5 i II, following 3 days of ALK5 i II treatment. Nanog and Oct4 also increased by approximately 3.7- and 5.6-fold, respectively, after 3 days of ALK5 i II treatment (Figure 6).

## 4. Discussion

ADSCs have been used in various fields of regenerative medicine because they can be harvested less invasively than other stem cells and have a high yield and rapid cell proliferation. With regard to peripheral nerve injury, evidence is gradually accumulating that SCLCs induced from ADSCs promote axonal regeneration and myelination. However, few reports have addressed SCs that are induced from human adipose tissue and maintain functions applicable to clinical practice. If such cells are available, cell transplantation therapy for peripheral nerve injury and neurodegenerative diseases may likely be possible using the ADSC-derived SCLCs.

By the treatment with ALK5 i II, ADSCs underwent a marked morphological change from fibroblast-like into spindle in shape, and obtained expression of typical SC markers, S100*β*, GAP43, EGR2, and NCAM, as demonstrated by RT-PCR and immunostaining analyses. Reportedly, human cSCLCs that were derived from ADSCs expressed typical markers such as S100*β* at only low levels [7], whereas mSCLCs induced by ALK5 i II did so at remarkably high levels under our experimental conditions. Moreover, the cells promoted axonal growth at a comparable level to gSCs. These results indicate that the mSCLCs have acquired a closer SC phenotype than cSCLCs and were functionally similar to gSCs.

Among the six TGF*β*R inhibitors that we tested, ALK5 i II most significantly elevated expression of S100*β*, GAP43, and EGR2 in ADSCs. In previous studies [27], SB431542 (SB) was frequently used as a TGF*β*R inhibitor, but we found that ALK5 i II was more potent than SB in converting ADSCs into SCLCs. ALK5 i II strongly inhibits Smad2/3 signaling in fibroblasts [14, 17, 28]. In our experimental settings, ALK5 i II prevented phosphorylation of Smad2/3 in ADSCs (Figure S3). ALK5 i II is a selective TGF*β*R1 inhibitor that blocks binding of ATP to TGF*β*R1 and subsequent TGF*β*R1 phosphorylation, thus inhibiting both Smad and non-Smad signaling pathways [14, 23]. In the Smad pathway, Smad2 and Smad3 are phosphorylated in response to TGF*β*R1 phosphorylation and associate with Smad4 to form complexes that subsequently migrate into the nucleus to exert regulatory functions [24]. ALK5 i II also induces conversion of human dermal fibroblasts into osteoblasts [17] and adipogenesis of mouse embryonic fibroblasts [18].

In an attempt to figure out the potential mechanisms underlying the ALK5 i II-mediated induction of mSCLCs, knockdown of Smad2 and/or Smad3 by shRNA did not influence the induction of the mSCLC phenotype in ADSCs, indicating that the non-Smad signaling may play crucial roles. Among the non-Smad pathways, JNK, p38, ERK, or MEK may not play crucial roles, because inhibitors of these pathways failed to augment phenotypic alteration of ADSCs into mSCLCs (Figure 2). Alternatively, two or more pathways might be involved in a cooperative manner.

Meanwhile, we found that Nanog, Oct4, and Sox2 were upregulated in hADSCs 3 days after supplementation of ALK5 i II (Figures 6 and S1). These are the transcription factors specifically expressed in pluripotent stem cells. Among others, Oct4, a member of the Oct family transcription factors, is used as the gene to induce pluripotency in somatic cells [29], while it is also known to cause direct conversion from human fibroblasts to osteoblasts [30, 31], adipocytes [32], and hepatocytes [32]. This suggests that Oct4 may induce epigenetic reprogramming to an immature state, which may be an important step of transdifferentiation. Another possible explanation is that Sox2, a Sox family member, was involved in transdifferentiation of ADSCs to mSCLCs and in promotion of cell proliferation. ALK5 i II is well known to replace exogenous Sox2 in induction of reprogramming and maintenance of pluripotency [14]. In the central nervous system, Sox2 is detected in neural stem cells from neurogenic regions and maintains stem cell proliferation and differentiation [33, 34]. In the peripheral nervous system, Sox2 is considered mainly to be expressed in immature or dedifferentiated Schwann cells [35], but more recent studies have identified specific types of more differentiated glia that retain high Sox2 expression and critically require Sox2 function, as revealed by functional studies in mice and other animals [36, 37]. It has also been found that overexpression of Sox2 in SCs enhances their proliferation [38]. Further studies are needed to address the molecular mechanisms of mSCLC induction in more detail.

Overall, we successfully induced ADSCs into mSCLCs without involvement of any exogenous gene transduction. The mSCLCs showed a more robust SC phenotype pattern than cSCLCs and were functionally more similar to gSCs. Thus, use of ALK5 i II is a more promising method for induction of ADSCs into SCLCs, as demonstrated by morphological, mRNA expression, and protein expression characteristics of the mSCLCs. These findings may lead to a novel autologous cell replacement strategy to generate mature and phenotypically stable SCs, which may contribute to the treatment of PNI and neurodegenerative neural diseases. The induction mechanism was suggested to involve the non-Smad pathway and enhancement of intracellular expression of Sox2, which is a known pluripotent stem cell marker and a partial SC marker, and Oct4. We are currently undertaking studies of the detailed molecular mechanisms underlying this process.

## 5. Conclusions

This study demonstrated that ALK5 i II can accelerate the induction of ADSCs toward SC-like phenotypic cells (mSCLCs) that expressed SC-specific genes more strongly than cSCLCs. Furthermore, mSCLCs displayed the neurosupportive capacity in vitro on the same level as gSCs. ALK5 i II exerts its effect through a non-Smad pathway and following upregulation of undifferentiated cell-related genes such as Sox2. This approach might be applicable to cell-based therapy for peripheral nerve injury.

## Data Availability

The supplementary data used to support the findings of this study are included within the supplementary information file.

## Abbreviations

SC: Schwann cell
SCLC: Schwann cell-like cell
cSCLC: Conventional Schwann cell-like cell
gSC: Genuine Schwann cell
mSCLC: Modified Schwann cell-like cell.

## Supplementary Materials

Supplementary Materials Table S1: list of compounds used in the study. Table S2: list of TGF*β* receptor inhibitors used in the study. Table S3: list of probes and primers used in the study. Table S4: ADSCs but not mSCLCs expressed ADSC markers at high levels. Figure S1: scheme of the experimental design. Figure S2: mSCLCs expressed S100*β*, GAP43, EGR2, and NCAM as revealed by immunostaining. Figure S3: an addition of ALK5 i II suppressed phosphorylation of Smad2/3 in ADSCs. Figure S4: inhibitory effect of shRNAs determined by real-time RT-PCR.

## References

[1] Sowa Y., Kishida T., Tomita K., Yamamoto K., Numajiri T., Mazda O. (2017). Direct conversion of human fibroblasts into Schwann cells that facilitate regeneration of injured peripheral nerve in vivo. *Stem Cells Translational Medicine*.

[2] Thoma E. C., Merkl C., Heckel T. (2014). Chemical conversion of human fibroblasts into functional Schwann cells. *Stem Cell Reports*.

[3] Kitada M., Murakami T., Wakao S., Li G., Dezawa M. (2019). Direct conversion of adult human skin fibroblasts into functional Schwann cells that achieve robust recovery of the severed peripheral nerve in rats. *Glia*.

[4] Kingham P. J., Kalbermatten D. F., Mahay D., Armstrong S. J., Wiberg M., Terenghi G. (2007). Adipose-derived stem cells differentiate into a Schwann cell phenotype and promote neurite outgrowth in vitro. *Experimental Neurology*.

[5] Di Summa P. G., Kingham P. J., Raffoul W., Wiberg M., Terenghi G., Kalbermatten D. F. (2010). Adipose-derived stem cells enhance peripheral nerve regeneration. *Journal of Plastic, Reconstructive & Aesthetic Surgery*.

[6] Tomita K., Madura T., Mantovani C., Terenghi G. (2012). Differentiated adipose-derived stem cells promote myelination and enhance functional recovery in a rat model of chronic denervation. *Journal of Neuroscience Research*.

[7] Tomita K., Madura T., Sakai Y., Yano K., Terenghi G., Hosokawa K. (2013). Glial differentiation of human adipose-derived stem cells: implications for cell-based transplantation therapy. *Neuroscience*.

[8] Spreca R. D. A., Rambotti M. G., Rende M., Saccardi C., Aisa M. C., Giambanco I. (1989). Immunocytochemical degenerating localization regenerating of S-bOb rat sciatic protein nerves. *The Journal of Histochemistry and Cytochemistry*.

[9] Rambotti R. D. M. G., Spreca A., Leoncini P., Estenoz M., Costantino-Ceccarini E., Giambanco I. (1990). Detection of S-100b protein in triton cytoskeletons: an immunocytochemical study on cultured Schwann cells. *The Journal of Histochemistry and Cytochemistry*.

[10] Donato R., Cannon B. R., Sorci G. (2013). Functions of S100 proteins. *Current Molecular Medicine*.

[11] Lin T., Ambasudhan R., Yuan X. (2009). A chemical platform for improved induction of human iPS cells. *Nature Methods*.

[12] Zhu S., Li W., Zhou H. (2010). Reprogramming of human primary somatic cells by OCT4 and chemical compounds. *Cell Stem Cell*.

[13] Yu J., Chau K. F., Vodyanik M. A., Jiang J., Jiang Y. (2011). Efficient feeder-free episomal reprogramming with small molecules. *PLoS One*.

[14] Ichida J. K., Blanchard J., Lam K. (2009). A small-molecule inhibitor of Tgf-*β* signaling replaces Sox2 in reprogramming by inducing Nanog. *Cell Stem Cell*.

[15] Li W., Zhou H., Abujarour R. (2009). Generation of human induced pluripotent stem cells in the absence of exogenous *Sox2*. *Stem Cells*.

[16] Lyssiotis C. A., Foreman R. K., Staerk J. (2009). Reprogramming of murine fibroblasts to induced pluripotent stem cells with chemical complementation of Klf4. *Proceedings of the National Academy of Sciences of the United States of America*.

[17] Yamamoto K., Kishida T., Nakai K. (2018). Direct phenotypic conversion of human fibroblasts into functional osteoblasts triggered by a blockade of the transforming growth factor-*β* signal. *Scientific Reports*.

[18] Tu W. Z., Fu Y. B., Xie X. (2019). RepSox, a small molecule inhibitor of the TGF*β* receptor, induces brown adipogenesis and browning of white adipocytes. *Acta Pharmacologica Sinica*.

[19] Morimoto Y., Kishida T., Kotani S.-i., Takayama K., Mazda O. (2018). Interferon-*β* signal may up-regulate PD-L1 expression through IRF9-dependent and independent pathways in lung cancer cells. *Biochemical and Biophysical Research Communications*.

[20] Qiao L. J., Kang K. L., Heo J. S. (2011). Simvastatin promotes osteogenic differentiation of mouse embryonic stem cells via canonical Wnt/*β*-catenin signaling. *Molecules and Cells*.

[21] Zhao Y., Zhao T., Guan J. (2015). A XEN-like state bridges somatic cells to pluripotency during chemical reprogramming. *Cell*.

[22] Jiang J. X. S., Choi R. C. Y., Siow N. L., Lee H. H. C., Wan D. C. C., Tsim K. W. K. (2003). Muscle induces neuronal expression of acetylcholinesterase in neuron-muscle co-culture: transcriptional regulation mediated by camp-dependent signaling. *The Journal of Biological Chemistry*.

[23] Valcourt U., Kowanetz M., Niimi H., Heldin C.-H., Moustakas A. (2005). TGF-*β* and the Smad signaling pathway support transcriptomic reprogramming during epithelial-mesenchymal cell transition. *Molecular Biology of the Cell*.

[24] Crane J. L., Cao X. (2014). Bone marrow mesenchymal stem cells and TGF-*β* signaling in bone remodeling. *The Journal of Clinical Investigation*.

[25] Choe C., Kim H., Min S., Park S., Seo J., Roh S. (2018). SOX2, a stemness gene, induces progression of NSCLC A549 cells toward anchorage-independent growth and chemoresistance to vinblastine. *Oncotargets and Therapy*.

[26] Zhao X. X., An X. L., Zhu X. C. (2018). Inhibiting transforming growth factor-*β* signaling regulates in vitro maintenance and differentiation of bovine bone marrow mesenchymal stem cells. *Journal of Experimental Zoology Part B: Molecular and Developmental Evolution*.

[27] Inman G. J., Nicolás F. J., Callahan J. F. (2002). SB-431542 is a potent and specific inhibitor of transforming growth factor-*β* superfamily type I activin receptor-like kinase (ALK) receptors ALK4, ALK5, and ALK7. *Molecular Pharmacology*.

[28] Esebanmen G. E., Langridge W. H. R. (2017). Mechanism of chimeric vaccine stimulation of indoleamine 2,3-dioxygenase biosynthesis in human dendritic cells is independent of TGF-*β* signaling. *Cellular Immunology*.

[29] Takahashi K., Yamanaka S. (2006). Induction of pluripotent stem cells from mouse embryonic and adult fibroblast cultures by defined factors. *Cell*.

[30] Yamamoto K., Kishida T., Sato Y. (2015). Direct conversion of human fibroblasts into functional osteoblasts by defined factors. *Proceedings of the National Academy of Sciences of the United States of America*.

[31] Mizoshiri N., Kishida T., Yamamoto K. (2015). Transduction of Oct6 or Oct9 gene concomitant with Myc family gene induced osteoblast-like phenotypic conversion in normal human fibroblasts. *Biochemical and Biophysical Research Communications*.

[32] Wu W., Jin Y. Q., Gao Z. (2017). Directly reprogramming fibroblasts into adipogenic, neurogenic and hepatogenic differentiation lineages by defined factors. *Experimental and Therapeutic Medicine*.

[33] Wakamatsu Y., Uchikawa M. (2021). The many faces of *Sox2* function in neural crest development. *Development, Growth & Differentiation*.

[34] Kioke T., Wakabayashi T., Mori T., Takamori Y., Hirahara Y., Yamada H. (2014). Sox2 in the adult rat sensory nervous system. *Histochemistry and Cell Biology*.

[35] Pevny L. H., Nicolis S. K. (2010). Sox2 roles in neural stem cells. *The International Journal of Biochemistry & Cell Biology*.

[36] Koike T., Wakabayashi T., Mori T., Hirahara Y., Yamada H. (2015). Sox2 promotes survival of satellite glial cells in vitro. *Biochemical and Biophysical Research Communications*.

[37] Mercurio S., Serra L., Nicolis S. K. (2019). More than just stem cells: functional roles of the transcription factor Sox2 in differentiated glia and neurons. *International Journal of Molecular Sciences*.

[38] Roberts S. L., Dun X. P., Doddrell R. D. S. (2017). Sox2 expression in Schwann cells inhibits myelination *in vivo* and induces influx of macrophages to the nerve. *Development*.

