# Corrigendum to “ALK5 i II Accelerates Induction of Adipose-Derived Stem Cells toward Schwann Cells through a Non-Smad Signaling Pathway”

**DOI:** 10.1155/2025/9813648

**Published:** 2025-05-26

S. Sawai, T. Kishida, S.-I. Kotani, et al., “ALK5 i II Accelerates Induction of Adipose-Derived Stem Cells toward Schwann Cells through a Non-Smad Signaling Pathway”, *Stem Cells International*, no. 1 (2021): 1–11. https://doi.org/10.1155/2021/8307797

In the article titled “ALK5 i II Accelerates Induction of Adipose-Derived Stem Cells toward Schwann Cells through a Non-Smad Signaling Pathway”, https://doi.org/10.1155/2021/8307797, there are minor errors in Figures 1, 2, 3, 4, 5, and 6. The corrected figures are as follows:

Additionally, there were errors in the Section 3.2. These errors are shown below, “Real-time RT-PCR revealed that mSCLCs expressed mRNA for S100*β*, GAP43, EGR2, and NCAM approximately 11-fold, 18-fold, and 3.4-fold higher than cSCLCs, respectively (Figure 3(a))” should read “Real-time RT-PCR revealed that mSCLCs expressed mRNA for S100*β*, GAP43, EGR2, and NCAM approximately 11-fold, 52-fold, 18-fold, and 3.4-fold higher than cSCLCs, respectively (Figure 3(a)).”

“Some mSCLCs displayed bipolar and tripolar morphologies and strongly expressed S100*β* and GFAP proteins.” should read “Some mSCLCs displayed bipolar and tripolar morphologies and strongly expressed S100*β* and GAP43 proteins.”

The authors apologize for these errors and confirm that it does not affect the results and the conclusions of the article.

## Figures and Tables

**Figure 1:**
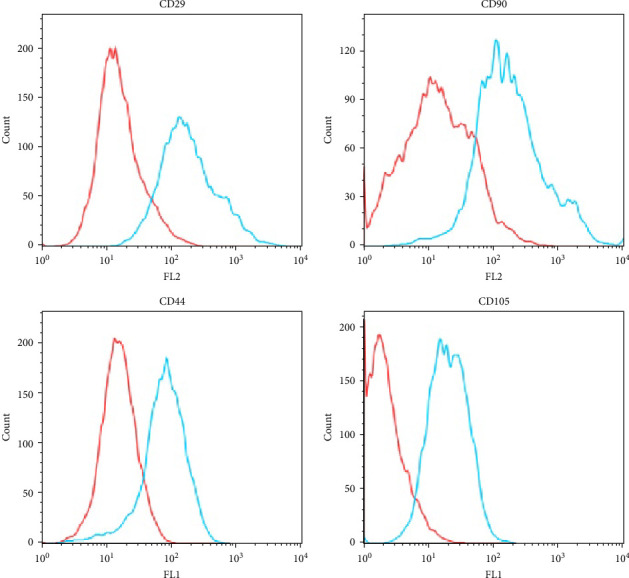
MSC markers decreased upon transition of ADSCs to mSCLCs. Human ADSCs were seeded onto 60 mm dishes and cultured in a complete medium or a SC medium supplemented with ALK5 i II for 14 days. Flow cytometric analysis was performed to examine CD29, CD44, CD90, and CD105 expression on the cell surface. Representative histograms for ADSCs (blue line) and mSCLCs (red line) are shown.

**Figure 2:**
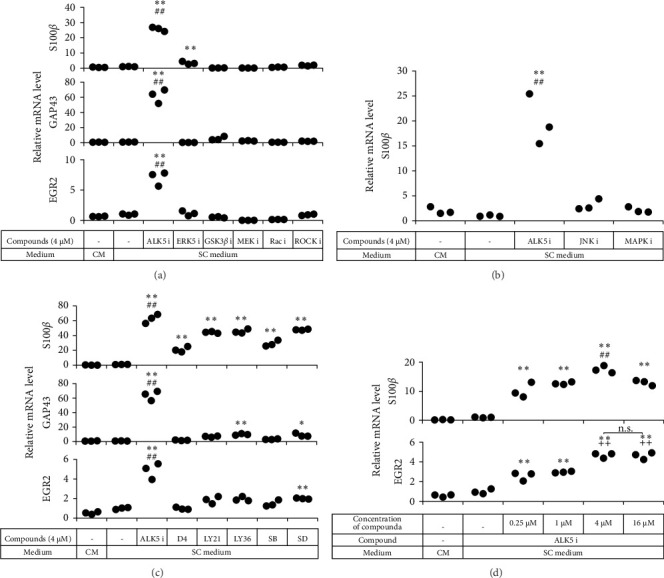
ALK5 i II prompted hADSCs to express SC markers. Human ADSCs were seeded in 24-well plates and cultured in a complete medium or a SC medium supplemented with the compounds indicated. Various signal pathway inhibitors (a, b) and TGF*β*R inhibitors (c) were compared, while various concentrations of ALK5 i II were also tested (d). After 14 days of culture, RNA was extracted from the cells and subjected to real-time RT-PCR analysis. Each dot represents triplicate value of relative mRNA level for the indicated genes. *∗p* < 0 : 05 and *∗∗p* < 0 : 01 vs. ADSCs cultured in the SC medium. ##*p* < 0 : 01 vs. all the other groups. ++*p* < 0 : 01 vs. ADSCs cultured in the SC medium supplemented with ALK5 i II at 0, 0.25, or 1 μM. n.s.: no significant difference between the indicated groups. Experiments were repeated three times.

**Figure 3:**
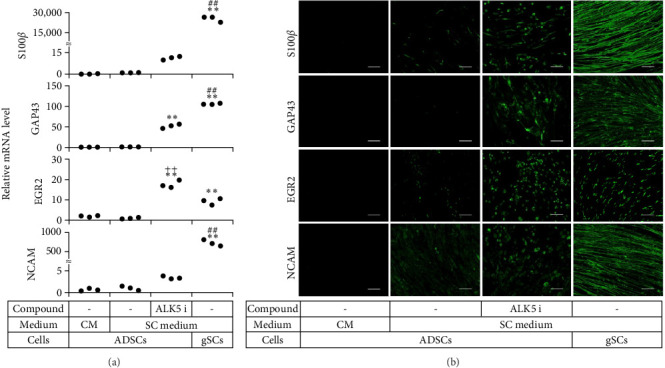
Comparison of relative mRNA levels among ADSCs, cSCLCs, mSCLCs, and gSCs. Cells were seeded into 24-well plates and cultured in the indicated medium for 14 days. (a) RNA was extracted from the cells and real-time RT-PCR was performed to evaluate mRNAs for the indicated genes. Each dot represents triplicate value. *∗∗p* < 0 : 01 vs. ADSCs. ##*p* < 0 : 01 vs. mSCLCs. ++*p* < 0 : 01 vs. gSCs. (b) ADSCs as negative control and cSCLCs, mSCLCs, and gSCs as positive control were stained with the indicated antibodies, while cell nuclei were stained with Hoechst. Fluorescence microscopic images (magnification: ×200) are shown. Scale bar = 100 μm. Experiments were repeated three times.

**Figure 4:**
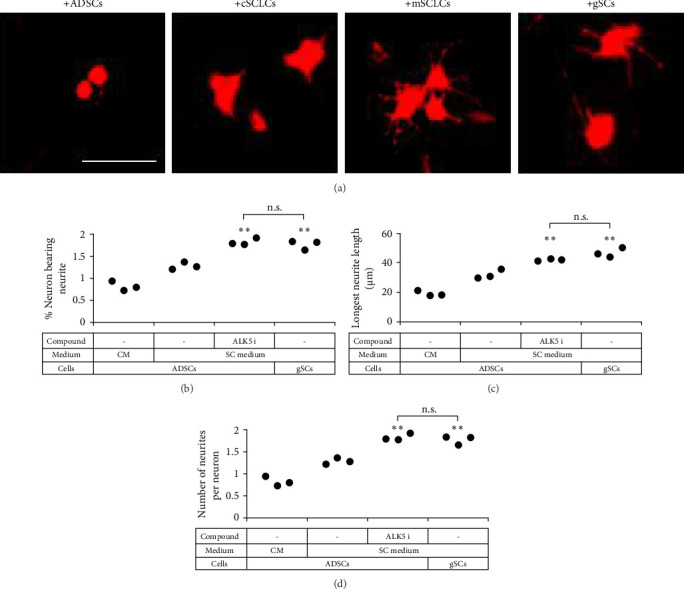
mSCLCs enhanced neurite outgrowth in NG108-15 cells. NG108-15 neuronal cells were cocultured with ADSCs (+ADSCs), cSCLCs (+cSCLCs), mSCLCs (+mSCLCs), or gSCs (+gSCs) for 24 h. NG108-15 cells were immunostained with MAP-2 antibody (red fluorescence) for neurite growth analysis. (a) Representative fluorescence microscopic images are shown. Scale bar = 100 μm. (b–d) Percentages of neurons bearing neurites (b), longest neurite lengths (c), and number of neurites per neuron (d) were calculated. Each dot represents a triplicate value. *∗∗p* < 0 : 01 vs. ADSCs cultured in a SC medium alone. n.s.: no significant difference between the indicated groups.

**Figure 5:**
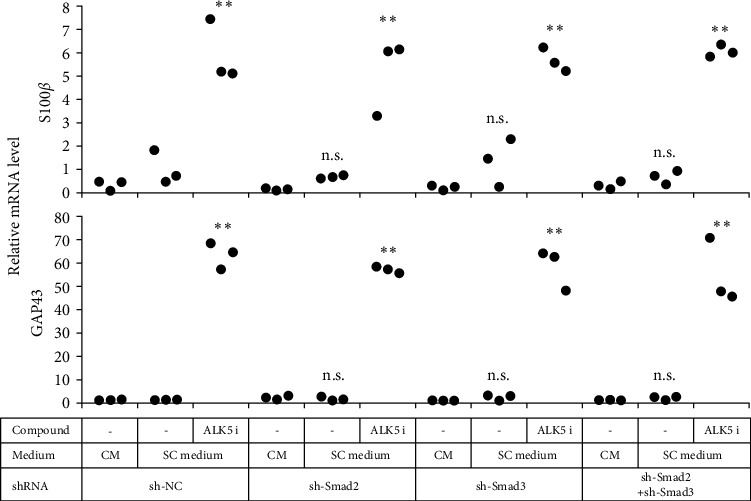
Neither Smad2 nor Smad3 were indispensable for induction of ADSCs into mSCLCs. ADSCs were transduced with lentiviral vectors encoding sh-NC, sh-Smad2, and/or sh-Smad3 as indicated. Forty-eight hours later, selection of transfected cells was started by adding puromycin and/or blasticidin. Stable transfectants were treated with BME and ATRA as indicated and cultured in the indicated culture medium as in Figure S1. After 14 days of culture, RNA was extracted from the cells and subjected to real-time RT-PCR to determine mRNA levels of the indicated genes. Each dot represents a triplicate value. *∗∗p* < 0 : 01 vs. sh-NC/SC medium/no compound. n.s.: no significant difference vs. sh-NC/SC medium/no compound.

**Figure 6:**
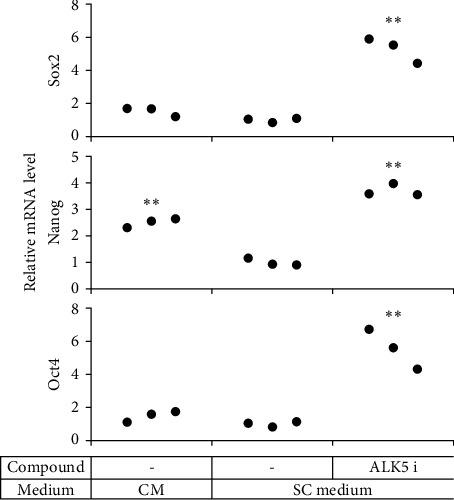
Impact of ALK5 i II on pluripotency-related gene expression in mSCLCs. ADSCs were cultured in a SC medium with ALK5 i II as in Figure 2. RNA was extracted from the cells 3 days after medium replacement (Figure S1) and subjected to realtime RT-PCR to evaluate mRNA levels for the indicated genes. Each dot represents a triplicate value of the relative mRNA level. *∗∗p* < 0 : 01 vs. ADSCs cultured in the SC medium alone.

