# Supplementary material for: ALK5 i II Accelerates Induction of Adipose-Derived Stem Cells toward Schwann Cells through a Non-Smad Signaling Pathway
Source: Stem Cells Int. 2021 Oct 15;2021:8307797. doi: 10.1155/2021/8307797 (PMC8536445; doi:10.1155/2021/8307797)
Supplement: Supplementary Materials — Table S1: list of compounds used in the study. Table S2: list of TGFβ receptor inhibitors used in the study. Table S3: list of probes and primers used in the study. Table S4: ADSCs but not mSCLCs expressed ADSC markers at high levels. Figure S1: scheme of the experimental design. Figure S2: mSCLCs expressed S100β, GAP43, EGR2, and NCAM as revealed by immunostaining. Figure S3: an addition of ALK5 i II suppressed phosphorylation of Smad2/3 in ADSCs. Figure S4: inhibitory effect of shRNAs determined by real-time RT-PCR. [file 8307797.f1.docx]

**Supplementary Information**

| Nomenclature | Molecular  Weight | Final  concentration | Source  (Provider,  Catalogue number) |
| --- | --- | --- | --- |

| ERK5 inhibitor / XMD8-92 | 588.58 | 4 μM | Chemscene CS-0245 |
| --- | --- | --- | --- |
| GSK-3 inhibitor / CHIR99021 | 465.34 | 4 μM | StemRD, CHIR-002 |
| MEK inhibitor / PD0325901 | 482.19 | 4 μM | Wako, 162-25291 |
| Rac1 inhibitor / NSC23766 | 530.96 | 4 μM | Wako, 512-38581 |
| ROCK inhibitor / Y27632 | 338.27 | 4 μM | Nacalai, 08945-84 |
| JNK inhibitor /  JNK inhibitor VIII | 356.4 | 4 μM | Cayman 894804-07-0 |
| P38 MAPK inhibitor /  BMS-582949 | 406.48 | 4 μM | Selleck S8124 |
| TGF-β receptor inhibitor /  ALK5 inhibitor II | 287.3 | 0.25, 1, 4, 16 μM | StemRD, ALK-010 |

**Table S1.** List of compounds used in the study.

| Nomenclature (Abbreviation) | Molecular  Weight | Final  concentration | Source  (Provider,  Catalogue number) |
| --- | --- | --- | --- |

| ALK5 inhibitor II (ALK5 i II) | 287.3 | 0.25, 1, 4, 16 μM | StemRD, ALK-010 |
| --- | --- | --- | --- |
| D4476 (D4) | 398.4 | 4 μM | Calbiochem, 218705 |
| LY2157229 (LY21) | 369.4 | 4 μM | Cayman, 15312 |
| LY364947 (LY36) | 272.3 | 4 μM | Cayman, 13341 |
| SB431542 (SB) | 550.5 | 4 μM | Wako, 192-16542 |
| SD208 (SD) | 352.8 | 4 μM | TOCRIS, 3269 |

**Table S2.** List of TGFβ receptor inhibitors used in the study.

| Target gene | Provider | Catalogue number |
| --- | --- | --- |
| Human EGR2 | Applied Biosystems | Hs00166165_m1 |

| Human GAP43 | Applied Biosystems | Hs00967138_m1 |
| --- | --- | --- |
| Human S100β | Applied Biosystems | Hs00902901_m1 |
| Human NCAM | Applied Biosystems | Hs00941830_m1 |
| Human Nanog | Applied Biosystems | Hs04399610_g1 |
| Human Oct4 | Applied Biosystems | Hs01895061_u1 |
| Human Sox2 | Applied Biosystems | Hs00602736_s1 |

**Table S3.** List of probes and primers used in the study.


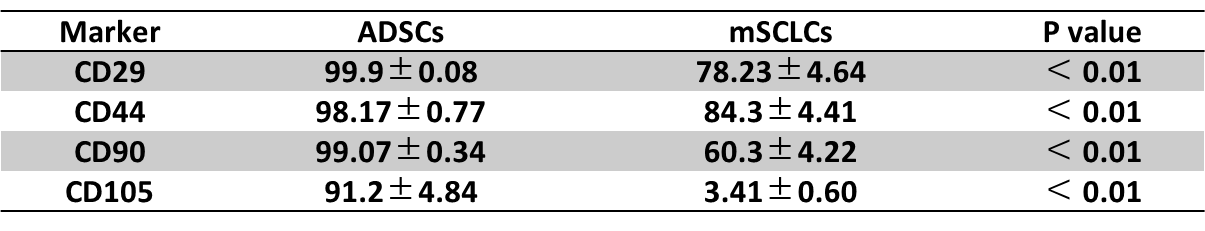


**Table S4. ADSCs but not mSCLCs expressed ADSC markers at high levels.**

Human ADSCs were seeded onto 60-mm dishes and cultured in complete medium or SC medium supplemented with ALK5 i II for 14 days. Flow cytometric analysis was performed to examine CD29, CD44, CD90, and CD105 expression on the cell-surface. Average +/- S.D. of percentage of positive cells are shown (N=3).


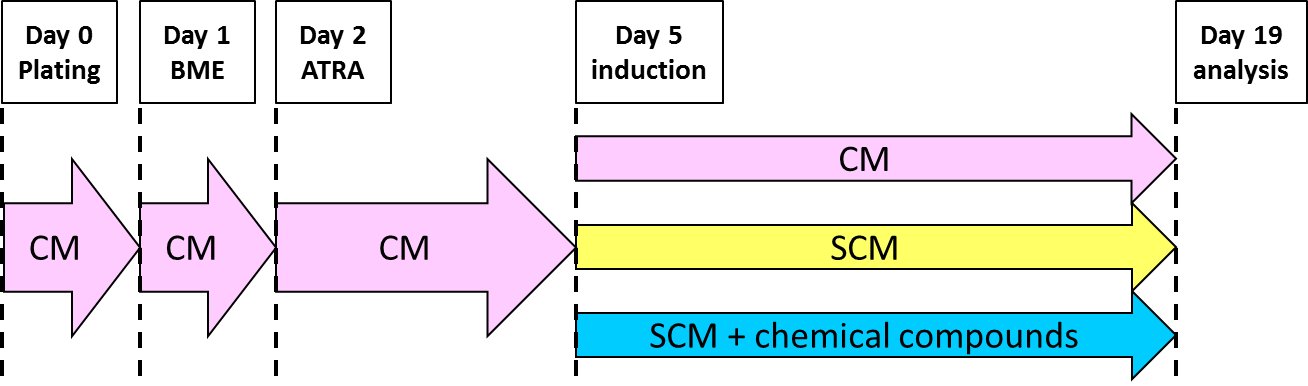


**Figure S1. Scheme of the experimental design.** CM: compete medium. SCM: Schwann cell medium.


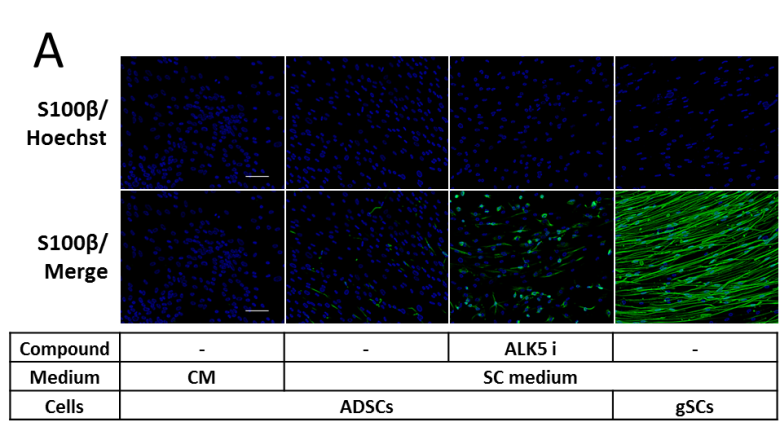


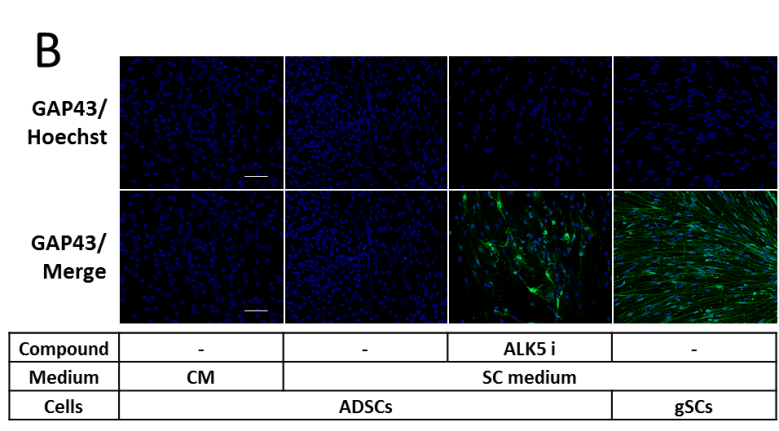


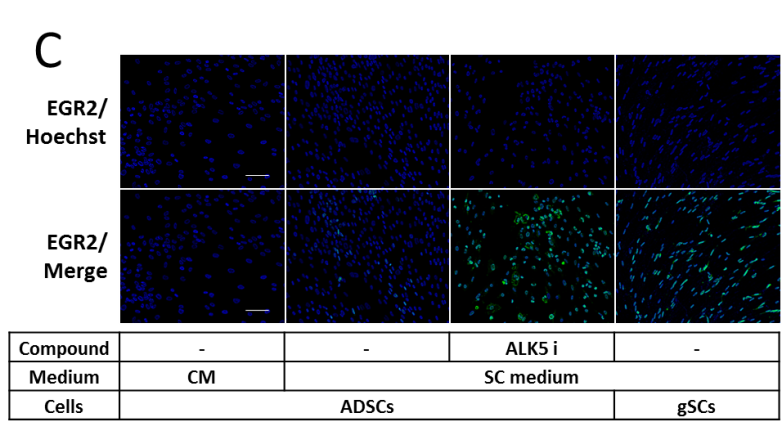


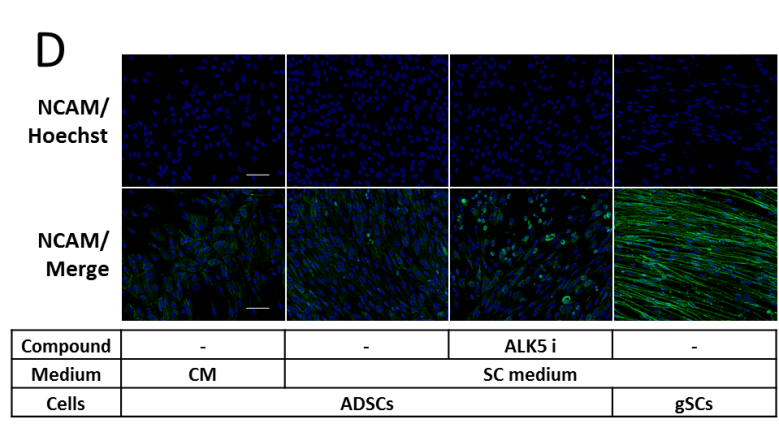


**Figure S2. mSCLCs expressed S100β, GAP43, EGR2 and NCAM as revealed by immunostaining.**

Cells were seeded into 24-well plates and cultured in the indicated medium for 14 days. Expression of S100β (A), GAP43 (B), EGR2 (C), and NCAM (D) proteins was detected by immunostaining. Representative fluorescence microscopic images (magnification ×200) are shown. Scale bar = 100 μm.


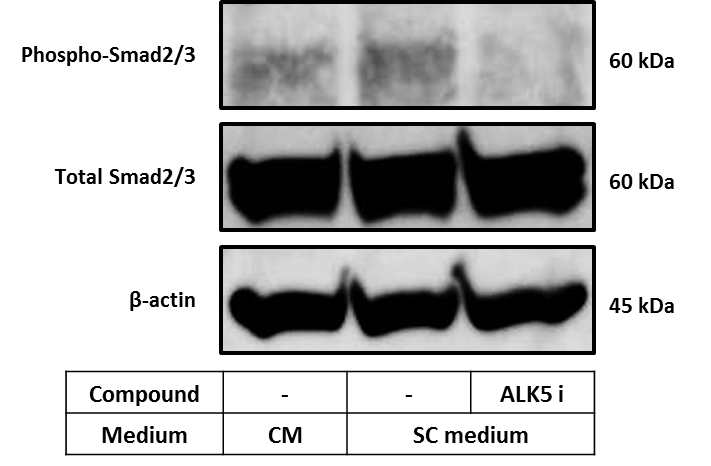


**Figure S3. An addition of ALK5 i II suppressed phosphorylation of Smad2/3 in ADSCs.**

Human ADSCs were seeded into 6-well plates and cultured in complete medium or SC medium supplemented with or without ALK5 i II. After culture for 2 days, culture supernatant was replaced by fresh one, and cells were lysed 30 min later. Western blotting analyses were performed using the indicated antibodies.


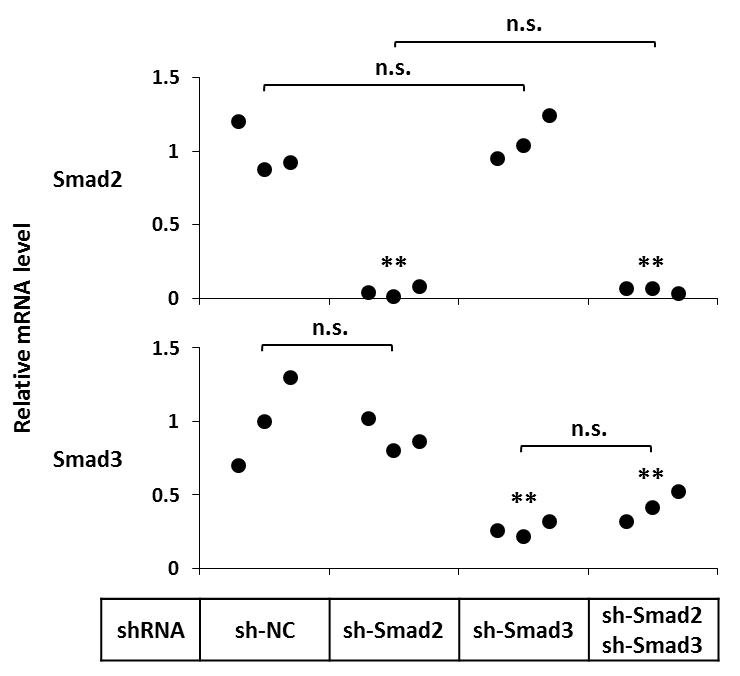


**Figure S4. Inhibitory effect of shRNAs determined by real time RT-PCR.**

Human ADSCs were seeded onto 60-mm dishes and transduced with lentiviral vectors encoding sh-NC, sh-Smad2 and/or sh-Smad3 as indicated. After the selection by puromycin (for sh-NC and sh-Smad2), blasticidin (for sh-Smad3), or both (for sh-Smad2/sh-Smad3), RNA was extracted from the cells and subjected to real time-RT-PCR analysis. Each dot shows relative mRNA level for Smad2 and Smad3. **P<0.01 vs. ADSCs transduced with sh-NC. n.s. No significant difference between the indicated groups.
